# Neurocognitive Impairment in HIV-Infected Naïve Patients with Advanced Disease: The Role of Virus and Intrathecal Immune Activation

**DOI:** 10.1155/2012/467154

**Published:** 2012-03-27

**Authors:** Monica Airoldi, Alessandra Bandera, Daria Trabattoni, Benedetta Tagliabue, Beatrice Arosio, Alessandro Soria, Veronica Rainone, Giuseppe Lapadula, Giorgio Annoni, Mario Clerici, Andrea Gori

**Affiliations:** ^1^Division of Infectious Diseases, Department of Internal Medicine, “San Gerardo” Hospital, University of Milan-Bicocca, 20900 Monza, Italy; ^2^LITA VIALBA, University of Milan, 20157 Milan, Italy; ^3^Department of Clinical Medicine and Prevention, University of Milan-Bicocca, SCC Geriatrics, 20900 Monza, Italy; ^4^Geriatric Unit, University of Milan, Fondazione Cà Granda, IRCCS Ospedale Maggiore Policlinico, 20122 Milan, Italy; ^5^Don C. Gnocchi Foundation, IRCCS, 20148 Milan, Italy

## Abstract

*Objective.* To investigate intrathecal immune activation parameters and HIV-RNA in HIV-associated neurocognitive disorders (HAND) of advanced naïve HIV-infected patients and to evaluate their dynamics before and after initiation of antiretroviral therapy (ART). *Methods.* Cross-sectional and longitudinal analysis of HIV RNA, proinflammatory cytokines (IL-6, IL-10, INF-**γ**, TNF-**α**, TGF-**β**1, and TGF-**β**2) and chemokines (MIP-1**α**, MIP-1**β**, and MCP-1) in plasma and cerebrospinal fluid (CSF) of HIV-infected patients with CD4 <200/**μ**L. *Results.* HAND was diagnosed at baseline in 6/12 patients. Baseline CSF HIV-RNA was comparable in patients with or without HAND, whereas CSF concentration of IL-6 and MIP-1**β**, proinflammatory cytokines, was increased in HAND patients. CSF evaluation at 12 weeks was available in 10/12 cases. ART greatly reduced HIV-RNA in all patients. Nevertheless, IL-6 and MIP-1**β** remained elevated after 12 weeks of therapy in HAND patients, in whom CSF HIV RNA decay was slower than the plasmatic one as well. *Conclusion.* Immune activation, as indicated by inflammatory cytokines, but not higher levels of HIV-RNA is observed in advanced naïve HIV-infected patients with HAND. In HAND patients, ART introduction resulted in a less rapid clearance of CSF viremia compared to plasma and no modifications of intratechal immune activation.

## 1. Introduction

HIV can cause a wide range of neurocognitive complications which were recently regrouped under the acronym of HAND, that is, HIV-associated neurocognitive disorders [1].

HAND reflects a spectrum of neurologic diseases ranging from subclinical neuropsychological impairment, such as asymptomatic neurocognitive impairment (ANI) or mild neurocognitive disorder (MND), to clinically evident HIV-associated dementia (HAD) [2]. Following the introduction of antiretroviral therapy (ART), the HAND profile has changed. Indeed, widespread use of ART has decreased the prevalence of HIV-associated dementia (HAD), but the prevalence of milder forms of HIV-associated neurocognitive disorders (HAND) has remained stable or increased among both treated and untreated individuals [3–5]. Prevalence of minor cognitive deficits has increased as well and is currently reported to be detectable in 20 to 50% of patients [6, 7]. The most probable explanation for this increase is that many antiretroviral agents exhibit poor CSF and brain penetration [8]; additionally, these agents, may fail to completely suppress HIV replication in the brain due to compartmentalization of HIV-1 infection within the central nervous system (CNS). 

HIV establishes an inflammatory response within the CNS, resulting in macrophage activation. The inflammatory response is considered to be the main mediator of neuronal damage in HIV-related neurodegenerative disease [9]. Despite effective ART, a substantial proportion of patients continue to show signs of macrophage/microglia activation and intratechal immunoglobulin production in the CNS [10].

The effects of ART on CNS viral burden and CNS immune activation need to be clarified in order to identify new strategies for the prevention and treatment of neurocognitive disorders.

In order to gain insight into these issues, we investigated the potential relation between CSF HIV RNA level, immune activation, and HAND in HIV-positive advanced *naïve* patients before and after starting ART. 

## 2. Material and Methods

### 2.1. Study Participants and Evaluations

Individuals were enrolled at the Infectious Diseases Unit of San Gerardo Hospital, University of Milan-Bicocca. Antiretroviral therapy-naive subjects with CD4+ <200 cells/*μ*L were eligible. Subjects with current or past CNS infections, psychiatric diseases, other chronic neurologic disorders, drugs or alcohol abuse were excluded. Written informed consent was obtained prior to enrolment.

At baseline and after 12 weeks of ART (zidovudine, lamivudine and lopinavir/ritonavir), patients underwent clinical examination, virological and immunological assessment, and CSF examination.

#### 2.1.1. Neuropsychological Evaluation

Neuropsychological evaluation was performed at baseline and at week 24. Neurocognitive function was assessed by a neuropsychological set of tests recommended by ACTG, designed to quantify cognitive impairment. These tests cover different cognitive domains: trail-making A (speed of processing); trail-making B (executive function); Mauri et al. test (Episodic memory); Matrix test (visual and selective attention, speed, and accuracy of visual searching); Wais-R vocabulary (language); Montgomery-Åsberg Depression Rating Scale (depressive symptoms). Performance on each test was normalized for age and education. ANI was defined as impairment in ≥2 cognitive domains that cannot be explained by opportunistic CNS disease, systemic illness, psychiatric illness, substance use disorders, or medications with CNS effects [2]. MND was defined as at least mild impairment (>1 SD below a demographically appropriate normative mean), involving ≥2 cognitive domains that cannot be explained by confounding conditions and reported or demonstrated mild functional decline that cannot be explained by confounding conditions [2].

#### 2.1.2. Laboratory Evaluation

HIV-RNA levels were measured in cell-free CSF and plasma using the PCR-Taqman (lower limit of detection 40 copies/mL). Cytokines (IL-6, IL-10, INF-*γ*, TNF-*α*, TGF-*β*1, TGF-*β*2) and chemokines (MIP-1*α*, MIP-1*β*, MCP-1) concentrations were assessed by ELISA (R&D System, Minneapolis, MN).

#### 2.1.3. Statistical Methods

Descriptive results are presented as medians with interquartile range (IQR). Chi-square or Fisher's exact test has been used to analyze categorical variables. ANOVA and Student's *t*-test were used for continuous variables unless they were abnormally distributed, in which case the Kruskal-Wallis and Mann-Whitney *U* tests were used.

All tests were two sided and a *P* value inferior to 0.05 was regarded as significant, although its meaning was merely descriptive.

## 3. Results

### 3.1. Patients Characteristics

Six advanced naïve HIV-infected patients with HAND (3 ANI and 3 MND) and 6 advanced naïve HIV+ patients without HAND (controls) were enrolled in the study (Table 1). Two patients were lost at followup in the HAND group (1 ANI and 1 MND). Median baseline CD4+ count was similar between the two groups: 88 cells/*μ*L (IQR 44–98) in HAND and 42 cells/*μ*L (IQR 42–74) in controls (*P* = 0.28). These values increased in both groups after initiation of ART, reaching 188 cell/*μ*L (IQR 178–205; *P* = 0.009) in HAND and 175 cell/*μ*L (IQ 123–394; *P* = 0.03) in controls at week 12.

### 3.2. Plasmatic and CSF HIV-RNA Dynamics

Plasmatic HIV RNA at baseline did not show statistically significant differences between the two groups of patients (HAND: 4.53 log copies/mL (IQR 4.22–5.55); controls: 5.30 log copies/mL (IQR 5.18–5.55) (*P* = 0.24)). Twelve weeks after starting ART, median HIV RNA in plasma was 2.32 log copies/mL (IQR 1.85–2.48) in HAND patients and 1.59 log copies/mL (IQR 1.59–1.72) in the control group. Median change in plasma HIV-RNA level at the end of followup was −3.07 log copies/mL (IQR −3.24–2.59) in HAND group and −3.60 (IQR −3.39–3.20) in the control group (*P* = 0.06).

**Figure 1:**
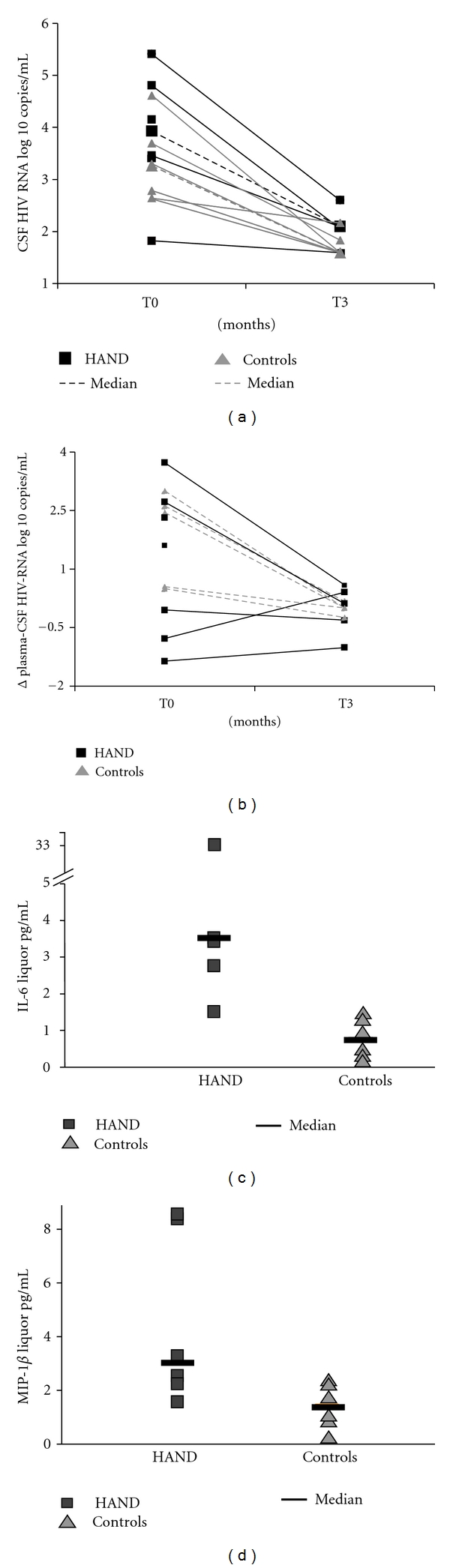
Representation of CSF HIV-RNA dynamics (a), difference in HIV-RNA levels between plasma and CSF (Δ) (b) and cytokines (IL-6, MIP-1*β*) concentrations at baseline. At baseline and after 12 weeks of ART, CSF HIV-RNA was not different between HAND patients and controls (Figure 1(a)). Difference in HIV-RNA levels between plasma and CSF (Δ), both at baseline and at week 12, showed a value > 0.5 log copies/mL in 3/4 patients in the HAND group and in 1/5 in the control group, reflecting a slower decrease in HIV-RNA level in CSF as compared to plasma in HAND patients (Figure 1(b)). At baseline concentration of MIP-1b and IL-6 in CSF, there was a difference between the two groups of patients (Figure 1(c)).

At baseline, median CSF HIV RNA was 3.61 log copies/mL (IQR 3.02–4.38) in the HAND group compared to 3.02 log copies/mL (IQR 3.39–4.64) in controls (*P* = 0.25). After 12 weeks of ART, median CSF HIV RNA was 2.09 log copies/mL in HAND group (IQR 1.59–2.08) and 1.59 log copies/mL (IQR 1.59–1.77) in the control group (Figure 1(a)). At the end of followup, median change of CSF HIV-RNA was −2.05 log copies/mL (IQR −2.76–1.08) in HAND group and −1.67 (IQR −1.86–1.19) in control group (*P* = 0.48).

We then calculated difference in HIV-RNA levels between plasma and CSF (Δ), both at baseline visit and at week 12. A value >0.5 log copies/mL was observed in 3 out of 4 patients in the HAND group and in 1/5 in the control group, reflecting a slower decrease in HIV-RNA level in CSF as compared to plasma in HAND patients (Figure 1(b)).

### 3.3. CSF Inflammation Dynamics

At baseline, concentration of inflammatory cytokines in CSF was different between the two groups of patients. Thus, MIP-1*β* was significantly elevated in HAND patients (2.74 pg/mL, IQR 2.14–7.30) compared to patients without HAND (1.44 pg/mL, IQR 0.94–2.13) (*P* = 0.03) (Figure 1(c)). Similarly, IL-6 CSF concentration was higher (3.5 pg/mL, IQR 2.96–6.22) in HAND patients compared to the control group (0.313 pg/mL IQR 0.18–1.04) (*P* = 0.06) (Figure 1(c)).

After 3 months of ART, no significant changes in cytokines and chemokines levels were observed in either group of patients despite a significant reduction in CSF HIV replication,. In particular, HAND patients did not show changes in the CSF concentration of either MIP-1*β* (2.65 pg/mL, IQR 1.42–1.87) or IL-6 (6 pg/mL, IQR 2.25–5.75) and concentrations of these cytokines thus remained significantly higher than controls.

CSF concentration of cytokines is chemokines are represented in Table 2. IL-10 and TNF-*α* CSF concentrations were undetectable in all patients at baseline and followup; INF-*γ* CSF concentration was detectable in 2/6 patients with HAND at baseline and 1/4 patients with HAND at week 12 (undetectable levels were found in controls at both timepoints).

## 4. Discussion

Data herein show that intrathecal immune activation plays a significant role in neuropathogenesis of cognitive impairment during HIV infection, as demonstrated in naïve patients with advanced immune suppression.

Typically, magnitude of CSF infection varies among patients. It does, however, correlate strongly with intratechal inflammation and development of HIV-associated dementia [9]. In our cohort of naïve HIV-infected patients with advanced disease, levels of HIV-RNA in blood and CSF of patients with HAND were comparable to those of patients without HAND. Interestingly, however, in HAND patients, we did observe a slower decay of CSF HIV replication as compared to plasma, indicating a more persistent replication of the virus in the CNS compartment. This suggests a model in which the presence of HAND is associated with compartmentalization of virus replication [7].

CNS immune activation accompanies HIV infection and is an important mediator of neurological injury. The specific mechanism(s) by which inflammatory cells are recruited into the CNS revolves around peripheral immune activation and a chemokine gradient established within the CNS as a result of viral infection and glial immune activation. In HAND patients, we found higher levels of macrophages-produced inflammatory cytokines, confirming the significant role of CNS macrophage activation in inducing the neurologic damage.

Currently, many unanswered questions remain regarding the relationship between neuroinflammation, blood-brain barrier (BBB) dysfunction, and progressive HIV-1 infection and how these factors affect the onset and development of HAND. The higher levels of IL-6 observed in our HAND patients confirm its specific role as mediator of neuroinflammation during chronic HIV inflammation. Recently, several studies identified possible pathways involved in the induction or generation of IL-6 from brain microvascular endothelial cells, as well as astrocytes [11, 12].

Moreover, higher levels of MIP-1*β* detected in the CSF of HIV-infected patients with HAND confirm previous observations of upregulation of this chemokine production in the brain of patients with ADC [13].

Similarly to what is observed for HIV-RNA levels, inflammation, as measured by CSF pleocytosis, decreases during ART-reduced CSF inflammatory response in the context of ART being suggested to be secondary to reduction of either local antigen stimulus or of HIV-related chemotaxis. Interestingly, in our advanced naïve HIV-infected HAND patients, intrathecal immune activation parameters, as measured by inflammatory cytokines is not influenced by ART initiation. This supports the hypothesis that intrathecal inflammation and immunoactivation play a critical role in patients with severe immune depression, associated with more frequent compartimentalization of HIV infection. Moreover, our observation confirms previous studies describing the persistence of CNS immune activation even in presence of virologically effective ART.

In our sample of advanced naïve HAND patients, the profile of CSF HIV infection was characterized by the presence of intratechal immune activation, as stated by the higher levels of inflammatory cytokines, accompanied by less rapid clearance of CSF viremia compared to plasma after the introduction of ART. Long-term observation focusing on neurological outcomes should provide further insight into the neurological prognosis of these patients.

## Figures and Tables

**Table 1:** Patients' characteristics.

						Baseline				Week 24			
Pt	Age	Sex	Ethnic Grup^a^	CDC	NPS Test^b^	CD4+ cell/*μ*L	HIV-RNA plasma log10 cp/mL	HIV-RNA CSF log10 cp/mL	CSF WBC cell/*μ*L	CD4+ cell/*μ*L	HIV-RNA plasma log10 cp/mL	HIV-RNA CSF log10 cp/mL	CSF WBC cell/*μ*L
1	40	M	C	C3	+	100	4.04	5.40	20	176	1.59	2.6	0
2	47	M	I	C3	+^b^	93	4.75	4.80	0	190	1.74	2.05	8
3	43	M	C	C3	+^b^	32	2.67	3.45	12	186	2.49	2.08	15
4	49	F	C	C3	+^b^	83	5.68	3.36	1	211	2.54		
5	59	M	C	C3	+	102	5.75	4.14	1	317	2.46		
6	27	M	I	C3	+	26	5.54	1.81	0	116	2.17	1.59	0
7	41	M	C	C3	−	42	5.87	3.26	0	234	1.76	1.59	0
8	36	M	C	B3	−	26	5.62	2.62	1	49	1.59	1.59	1
9	25	M	A	C3	−	172	5.16	4.16	0	145	1.59	1.59	0
10	25	F	I	B3	−	106	4.18	3.69	8	474	1.59	1.83	1
11	37	M	C	C3	−	148	5.36	2.63	0	448	2.27	2.16	0
12	53	M	C	C3	−	43	5.23	2.78	0	116	1.59	1.59	11

^a^: C: Caucasian, A: African, and I: Ispanic.

^b^: MND, +: altered NPS test.

**Table 2:** CSF inflammation markers dynamics.

CSF concentration (pg/mL)	HAND group	Controls	*P*
IL-6 BL	3.50 (2.96–6.22)	0.31 (0.18–1.04)	0.06
IL-6 W12	6.00 (2.25–5.75)	1.00 (0.00–1.52)	0.06
TGF-*β*1 BL	0.11 (0.00–11.75)	12.40 (0.00–24.10)	ns
TGF-*β*1 W12	0.10 (0.00–5.20)	13.50 (9.25–16.75)	ns
TGF-*β*2 BL	59.00 (0.00–87.20)	99.12 (20.10–147.00)	ns
TGF-*β*2 W12	46.11 (41.72–61.11)	79.06 (69.13–92.23)	ns
MIP-1*α* BL	0.70 (0.00–2.86)	0.10 (0.00–0.12)	0.05
MIP-1*α* W12	1.00 (1.00–6.50)	0.10 (0.00–0.22)	0.05
MIP-1*β* BL	2.74 (2.14–7.30)	1.44 (0.94–2.13)	0.03
MIP-1*β* W12	2.65 (1.42–1.87)	1.61 (0.48–1.87)	0.05
MCP-1 log10 BL	2.85 (2.84–3.06)	2.69 (2.65–2.75)	0.06
MCP-1 log10 W12	2.63 (2.61–2.79)	2.64 (2.61–2.68)	ns
